# Benthic and Pelagic Pathways of Methylmercury Bioaccumulation in Estuarine Food Webs of the Northeast United States

**DOI:** 10.1371/journal.pone.0089305

**Published:** 2014-02-18

**Authors:** Celia Y. Chen, Mark E. Borsuk, Deenie M. Bugge, Terill Hollweg, Prentiss H. Balcom, Darren M. Ward, Jason Williams, Robert P. Mason

**Affiliations:** 1 Dartmouth College, Department of Biological Sciences, Hanover, New Hampshire, United States of America; 2 Dartmouth College, Thayer School of Engineering, Hanover, New Hampshire, United States of America; 3 Stratus Consulting, Boulder, Colorado, United States of America; 4 University of Connecticut, Department of Marine Science, Groton, Connecticut, United States of America; 5 Humboldt State University, Department of Fisheries Biology, Arcata, California, United States of America; 6 Washington State University, Department of Civil and Environmental Engineering, Pullman, Washington, United States of America; Northwest Fisheries Science Center, NOAA Fisheries, United States of America

## Abstract

Methylmercury (MeHg) is a contaminant of global concern that bioaccumulates and bioamagnifies in marine food webs. Lower trophic level fauna are important conduits of MeHg from sediment and water to estuarine and coastal fish harvested for human consumption. However, the sources and pathways of MeHg to these coastal fisheries are poorly known particularly the potential for transfer of MeHg from the sediment to biotic compartments. Across a broad gradient of human land impacts, we analyzed MeHg concentrations in food webs at ten estuarine sites in the Northeast US (from the Hackensack Meadowlands, NJ to the Gulf of Maine). MeHg concentrations in water column particulate material, but not in sediments, were predictive of MeHg concentrations in fish (killifish and Atlantic silversides). Moreover, MeHg concentrations were higher in pelagic fauna than in benthic-feeding fauna suggesting that MeHg delivery to the water column from methylation sites from within or outside of the estuary may be an important driver of MeHg bioaccumulation in estuarine pelagic food webs. In contrast, bulk sediment MeHg concentrations were only predictive of concentrations of MeHg in the infaunal worms. Our results across a broad gradient of sites demonstrate that the pathways of MeHg to lower trophic level estuarine organisms are distinctly different between benthic deposit feeders and forage fish. Thus, even in systems with contaminated sediments, transfer of MeHg into estuarine food webs maybe driven more by the efficiency of processes that determine MeHg input and bioavailability in the water column.

## Introduction

Mercury (Hg) exposure from fish and seafood consumption results in both human and wildlife health effects [1]–[5]. However, while much of the research on food web processes influencing Hg concentrations in fish has been conducted in freshwater systems, approximately 90% of fish consumed in the US comes from estuarine and marine systems [6]. In these marine ecosystems, transformation of Hg to its most biologically toxic and available form, methylmercury (MeHg), is thought to occur primarily in estuarine and coastal sediments [7]–[9] where contaminant concentrations are highest, and in the oxygen minimum zone in the open ocean [10]. However, while MeHg in estuaries is produced *in situ* in sediments, it also comes from external sources such as ocean and watershed sources [11], [12]. Estuarine food webs therefore potentially provide important links between MeHg contamination in estuarine sediments and coastal fish and shellfish species, which constitute an important fraction of seafood consumed by humans and wildlife [13], [14].

It is well known that MeHg is accumulated by primary producers and other organisms at the base of aquatic food webs and biomagnifies during trophic transfer [15], [16]. The MeHg formed in sediments can enter the benthic food web directly through deposit feeding in the sediments by benthic infauna, which can then be consumed by predatory fish [17]. Additionally, it can also be directly transferred into the water column via advection and diffusion from sediments, or as a result of desorption from resuspended sediments [11], [18]. Water column MeHg can also be derived from upstream or offshore sources of dissolved and particulate MeHg [12], [19], [20]. Once in the water column, MeHg can be taken up by the pelagic food web.

Past research in freshwater systems has suggested that Hg bioaccumulation is a “bottom up” process driven by production or availability of aqueous MeHg, thus indicating that the net flux of MeHg from sediments to the water columns is extremely important [21], [22]. In coastal marine systems where watershed releases and offshore ocean sources of MeHg can be significant, the relative importance of bioaccumulation of MeHg from benthic vs. pelagic sources is not well understood. Various estuarine mass balance studies have suggested that the relative importance of sediment MeHg inputs varies from being a minor component of the total inputs (Bay of Fundy, Gulf of Maine and Hudson River estuary) [[12], [19], [20], [23], [24], to being one of the major sources (Chesapeake Bay, San Francisco Bay and Long Island Sound) [23],[25],[26],[27],[28]. Our recent reassessment of MeHg inputs to coastal ecosystems suggests that the importance of sediment sources of MeHg may have been overstated for some of these ecosystems [29]. The results from this study may therefore help reconcile the relative importance of sources of MeHg given that the study sampled locations in areas where previous mass balance studies have been completed (Hudson River, Long Island Sound and Gulf of Maine).

Past research has found that pelagic organisms bioaccumulate higher concentrations of MeHg than benthic fauna, suggesting that aqueous concentrations may be more important than sediment concentrations in determining the concentrations in higher trophic levels [30]. However, broad spatial studies also show concentrations in biota to be related to sediment concentrations [30]–[32]. There is still much to be learned about the movement of MeHg in coastal ecosystems from sediment and aqueous compartments to lower trophic level organisms that may be conduits of MeHg to fish species consumed by humans.

In both sediments and the water column, MeHg bioavailability is controlled by many factors including concentrations of total Hg, organic matter, and sulfide [11], [33] which determine the bioavailable MeHg concentration. In freshwater systems, binding of Hg and MeHg to dissolved organic carbon (DOC) can reduce bioavailability of Hg and MeHg to phytoplankton [34], [35] or may increase uptake when phytoplankton actively take up DOC that is bound to Hg and MeHg [36]. In estuarine sediments, organic matter is also important in controlling the distribution and bioavailability of Hg and MeHg, can either reduce or increase the amount of Hg methylation, and can reduce the bioavailability of MeHg to benthic organisms [11], [37]–[39]. The potential role of organic matter in controlling Hg and MeHg bioavailability in the water column and sediments is complex and may not simply depend on quantity but also organic matter quality [40].

In this study, we examine the distinct relationships between aqueous or sediment MeHg and bioaccumulation in common estuarine biota in coastal marshes, as well as the relationship of organic carbon in water (DOC) and sediments (total organic carbon or TOC) to total mercury in sediment and aqueous compartments. Coastal marshes are areas of direct Hg and MeHg watershed inputs and Hg methylation in sediments, and their food webs supply nutrition for estuarine and coastal fisheries resulting in a bioadvection of MeHg from coastal margins to the open ocean [13], [14], [41]. However, little is known about the pathways of MeHg bioaccumulation in these tidal systems where organisms feed in and on the sediments as well as in the water column. Identifying the sources of MeHg to these food webs is important ecologically as well as for regulatory and remediation purposes. Here, we investigated MeHg concentrations in estuarine fauna with different feeding modes across a broad range of sites from contaminated to pristine and addressed the following questions: 1) Do food sources (sediment vs. water column) influence bioaccumulation of MeHg in estuarine fauna? and 2) Does THg or MeHg in sediments relate directly to water column concentrations and does organic carbon influence those relationships? The overall goal of the study was to better understand the role of water column vs. sediment pathways in transferring MeHg to higher trophic level organisms, as well as the potential role of carbon in mediating MeHg bioavailability.

## Methods

### Ethics Statement

All the animal work conducted in this study were in compliance with Institutional Animal Care and Use Committee of the Geisel School of Medicine at Dartmouth College using protocols to minimize suffering of marine organisms collected and euthanized for tissue analysis.

**Figure 1:**
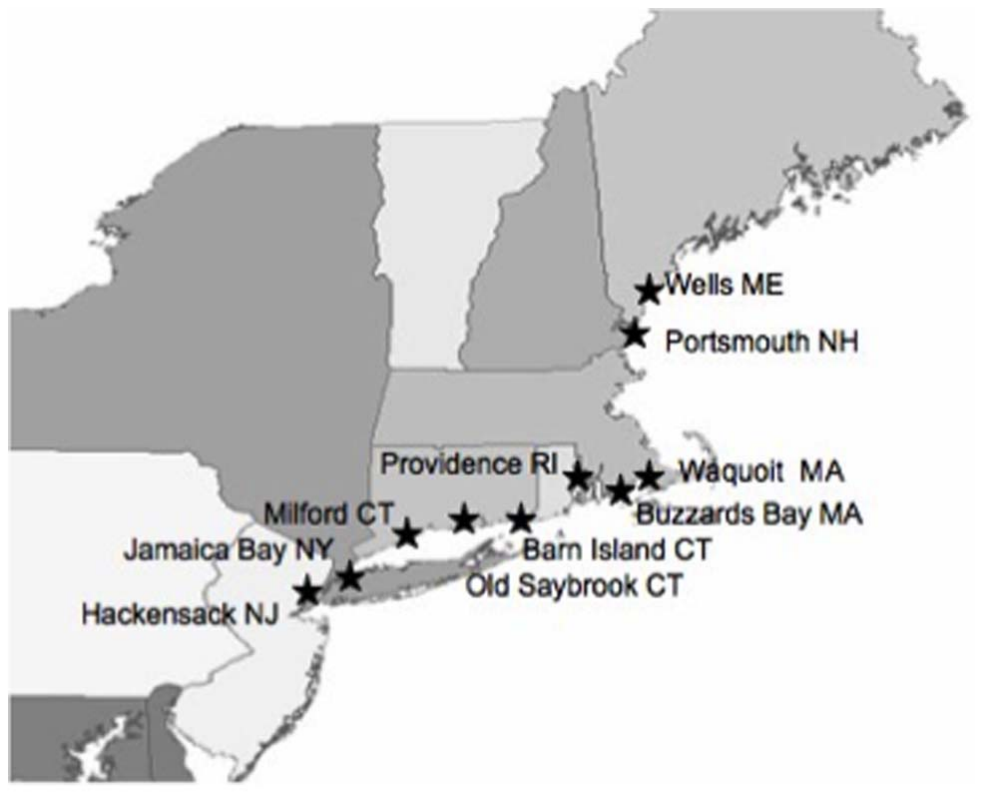
Map of field sites. Ten estuarine field sites in seven states (across a contamination gradient) sampled in summer 2008.

We collected biotic, water, and sediment samples at ten estuarine sites on the East Coast of the United States (from the Hackensack Meadowlands, NJ to the Gulf of Maine) along a human impact gradient (Figure 1). All field sites accessed were on public (municipal boat launches, Golden Gate National Park NY, Wells National Estuarine Research Reserve ME, Waquoit National Estuarine Research Reserve, Barn Island Wildlife Management Area CT) or private land (Milford Audubon Society CT, Indian Point Boat Yard MA) where permission to access the coastal marsh was obtained. The least contaminated and least developed site was the Webhannet Estuary in Wells, ME. The two most contaminated sites were the highly urbanized estuary of Jamaica Bay, NY, which has highly elevated metal levels relative to offshore waters [42], and Hackensack Meadowlands, NJ, where sediments are known to be contaminated with Hg through many years of heavy industrial and residential development, including the operation of a mercury recovery plant [43], [44]. Biotic, sediment and water column samples were collected in July–August 2008 and we measured inorganic Hg and MeHg in estuarine organisms, bulk sediments, filtered water, and suspended particulates. Biota included worms (polychaetes and oligochaetes), green crabs (*Carcincus maenas*), mussels (*Geukensia demissa)* at all sites except for *Mytilus edulis* in Portsmouth Harbor NH and Webhannet Estuary ME), killifish (*Fundulus heteroclitus*) and Atlantic silversides (*Menidia menidia*).

### Sediment and Water Sampling

Water sampling followed trace metal “clean” techniques, as outlined in Gill and Fitzgerald [45]. Water samples were collected at high tide off of a small fiberglass boat outfitted with an electric trolling motor. Boat surfaces were washed with water to remove any particulate material before entering the water. Water was collected with a peristaltic pump and C-flex tubing that had been soaked in 10% trace-metal grade hydrochloric acid (HCl) overnight and flushed with sample water prior to use. Surface water samples (one sample per site) were collected approximately 0.2 m below the surface. MeHg and total Hg water samples were filtered (600 to 750 ml) through in-line acid-cleaned Teflon filter holders fitted with pre-combusted quartz fiber filters into separate acid-cleaned Teflon bottles. These samples were acidified to 0.5% with trace-metal grade HCl, refrigerated, and stored in the dark until analysis. Water was also filtered (500 to 1050 ml) for DOC and total suspended solids (TSS) using pre-weighed and pre-combusted glass fiber filters. DOC samples were stored in acid-cleaned and pre-combusted glass vials with acid-cleaned Teflon lined caps, and frozen until analysis. Pre-weighed TSS filters were packaged in aluminum foil and frozen until analysis. Quartz fiber filters with samples for Hg species were stored frozen in acid-cleaned plastic petri dishes until particulate MeHg and total Hg analysis.

Sediment samples were collected at low tide in areas with at least 5 cm of water above the sediment surface. Two sediment cores at each site were taken using clear acid cleaned polycarbonate tubes (4.8 cm inner diameter). Cores were capped with 5 to 10 cm of overlying water and processed inside a nitrogen-filled glove bag within 2 hours after sampling. In the glove bag, overlying water was removed with a pipettor and 6 cm of sediment was homogenized and frozen until analysis.

### Biotic Sampling

Invertebrates were collected with plastic-coated minnow traps, D-nets, plastic and nylon sieves, or by hand. Killifish and Atlantic silversides were collected using both minnow traps and hand-drawn seines. All field collections of vertebrates were carried out in strict accordance with the recommendations of the veterinarians at the Animal Resource Center of the Geisel School of Medicine at Dartmouth College and the protocol approved by the Institutional Animal Care and Use Committee of the Geisel School of Medicine at Dartmouth College (Protocol # 07-03-03). Permits for collecting animal samples were obtained from Maine Department of Marine Resources, NH Fish and Game Department, MA Department of Division of Marine Fisheries, RI Department of Environmental Management, CT Department of Environmental Protection, NY State Department of Environmental Conservation, NJ Division of Fish and Wildlife, and the National Park Service. No protected species were collected.

All biotic samples were handled using trace metal clean techniques. Samples were either placed directly into acid-rinsed plastic ziptop bags or in acid-cleaned Teflon containers. Bags and containers were then double-bagged and samples were frozen until analysis. In the laboratory, frozen samples were thawed, rinsed in ultraclean water, blotted dry, and wet weight and total length or carapace width and length were recorded. Mussels were removed from their shells prior to freeze-drying. Organisms were transferred to new I-Chem Certified 300 Series vials and freeze dried (Labconco, FreeZone). Once dry, size class was determined based on length and dry weight and similar sized organisms from each site (n = 3 individuals) were selected across all field sites. Selected freeze-dried samples were ground and homogenized either with a ball mill equipped with Teflon grinding jars and balls (Retsch, Mixer Mill MM 301) or by hand using ceramic scissors (Kyocera) and a ceramic mortar and pestle.

### Water and Sediment Analysis

Sediment and water samples were analyzed at the University of Connecticut Department of Marine Sciences. Sediment percent loss on ignition (% LOI) was determined by combusting a subsample of freeze-dried sediment at 550°C for 8 hours. Concentrations of duplicate sediment samples per site were averaged for data analysis. MeHg in sediment was analyzed using a Perkin Elmer ELAN DRC II Inductively Coupled Plasma Mass Spectrometer (ICP-MS) following aqueous distillation (1 ml 50% H_2_SO_4_ and 0.5 ml 20% KCl), ethylation, gas chromatographic separation, and pyrolysis [46]–[49]. Concentrations of MeHg were calculated using external aqueous standards calibrated against a Hg standard traceable back to US National Institute of Standards and Technology (NIST) standard reference materials. For sediment MeHg, the RSD for field replicates was 19%, the recovery for samples spiked in the range of sample concentrations was 100±10% (mean ±SD), recovery of an estuarine sediment CRM (IAEA-405, IAEA) averaged 90%, and the detection limit (DL) was 0.003 ng g^−1^ [23]. Measurement of sediment total Hg was done using a DMA80 direct mercury analyzer [50]. Average recovery of a standard reference material (MESS-2, NRCC; estuarine sediment) was within the certified range (91±9 ng g^−1^) and the relative percent difference (RPD) between duplicate measurements (n = 7) was <5%.

Dissolved organic carbon (DOC) in water samples was measured with a Shimadzu TOC analyzer. MeHg analysis of filtered and particulate water samples was done as described above for sediment, but used cold vapor atomic fluorescence spectrometry detection (CVAFS; Tekran 2500). Filtered and particulate total Hg analysis was conducted following digestion, reduction, and purge and trap techniques detailed elsewhere [45], [51]–[54]. Concentrations of THg were calculated using an external aqueous standard (traceable to NIST) calibrated against an elemental mercury (Hg^0^) standard. Recovery for samples spiked in the range of sample concentrations averaged 112% for THg and 88% for MeHg, the relative standard deviation (RSD) of laboratory replicates was 11% for THg, and the DL was estimated to be 0.002 ng L^−1^ for MeHg analysis and 0.05 ng L^−1^ for THg (pore water) analysis. Water column particulate THg and MeHg concentrations (ng/L) were normalized to TSS to calculate suspended particle concentrations (ng/g).

### Biotic Hg and MeHg Analysis

Biotic samples were analyzed at the Trace Element Analysis Laboratory, Dartmouth College. For determination of THg and MeHg, samples were freeze-dried, spiked with an appropriate amount of enriched inorganic ^199^Hg (HgI) and enriched Me^201^Hg (MeHg) and then extracted in 2–3 ml of TMAOH (tetramethyl ammonium hydroxide, 25% w/v) [55]. MeHg and HgI were determined by species-specific isotope dilution purge and trap ICP-MS. Total Hg was calculated as the sum of MeHg and HgI [55], [56]. Three standard reference materials (SRMs) were used as quality control: NRCC DORM-3 dogfish mussel certified at 355±56 ng/g MeHg; 382±60 ng/g THg, NRCC TORT-2 lobster hepatopancreas certified at 152±13 ng/g and 270±60 ng/g THg, and NIST-2976 mussel tissue certified at 28±0.31 ng/g MeHg and 61±3.6 ng/g THg. Average recovery in DORM-3 was 92% (n = 7, rsd = 5.8%) for MeHg and 95% for THg (n = 7, rsd = 5.4%), 100% (n = 2, rsd = 2.3%) for MeHg and 113% for THg (n = 2, rsd = 12.3%) in TORT-2, and 104% (n = 7, rsd = 7.7%) for MeHg and 106% (n = 7, rsd = 25.2%) for THg in NIST-2976. The high variability in THg in the NIST-2976 is attributable to inorganic Hg being close to the detection limit in the SRM. The method detection limit for all three SRMs and the samples is 2 ng/g based on an initial sample weight of 25 mg.

### Stable Isotope Analysis

Approximately 1 mg of homogenous powder of each organism was analyzed for stable isotope ratios (^13^C/^12^C, ^15^N/^14^N) at the Stable Isotope Laboratory, Dartmouth College. Samples were flash combusted at >1020°C using a Carlo Erba elemental analyzer. The produced gases were carried by helium through a reduction column, a gas chromatography column and into a Conflo II unit, which subsamples the helium stream for input into an Advantage isotope ratio mass spectrometer (Thermo-Finnigan). Isotope ratios were corrected with three in-house standards ranging in C and N isotope ratios that have been calibrated against international standards. A set of standards was run for every 10 samples. δ^15^N was used to identify the trophic level of a given organism [57] while δ^13^C was used to identify whether an organism used benthic or pelagic food sources [58], [59].

### Data Analysis

Relationships between continuous variables were assessed by simple and multiple linear regression, after logarithmic transformations to ensure compliance with the usual regression assumptions. Linear regression analysis was used to compare concentrations of total Hg and MeHg in sediments and water (filtered and suspended particles) to total organic matter as %LOI in sediments and DOC in the water column. Multiple regression was used to determine the relationship between sediment and water column compartments of MeHg (sediment, filtered water, and particulates) and the various organisms (worms, mussels, green crabs, killifish, and Atlantic silversides). Tests of significance were conducted using sites, rather than individuals, as independent observations to avoid pseudoreplication [60]. Full models were first fit using concentrations in all sediment and aqueous compartments as predictors. Predictors with the highest p-values were then sequentially dropped until all predictors were significant, resulting in our reduced models. Regression results were used to construct a relational graph linking the Hg and MeHg concentrations in sediment and aqueous compartments with biotic compartments, using labels on each arrow to denote the relative contribution of each independent variable to the prediction of each dependent variable. These relative contributions were calculated by multiplying linear regression coefficients by the ratio of standard deviations of the independent and dependent variables, respectively. Relationships of MeHg in biota to food source (δ^13^C) and trophic level (δ^15^N) were also analyzed using linear regression analysis. δ^15^N values were adjusted for site by subtracting the corresponding site effects estimated from a two-way ANOVA with δ^15^N as the response variable and site and species as the treatment factors. All data analysis and production of figures were performed in R, an open-source statistical programming and graphics environment [61].

## Results

### Sediment and Water

**Figure 2:**
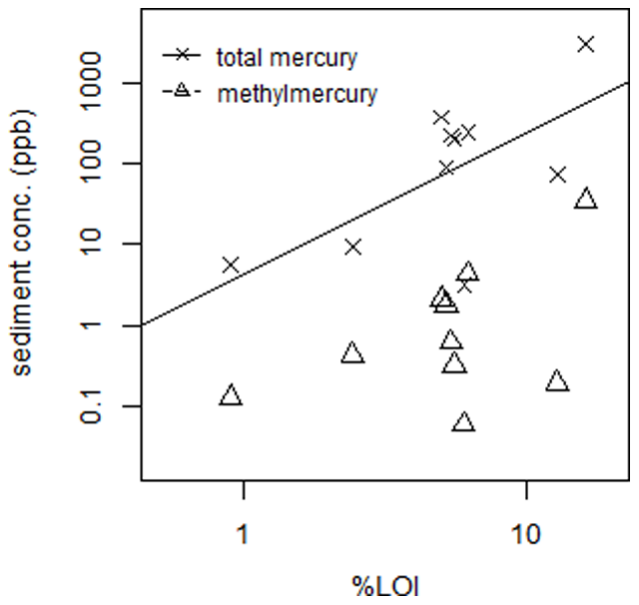
Relationships between sediment and water column compartments and organic carbon at each site. Relationships between continuous variables assessed by linear regression after logarithmic transformations. (A) total and MeHg sediment concentrations vs. %LOI.

Sediments from the ten sites sampled in this study ranged over two orders of magnitude in MeHg concentrations (0.13–34.8 ng/g dry wt.) and by a factor of 300 in total Hg (Table S1 in File S1). Sediment %MeHg also ranged widely, by more than an order of magnitude (Table S1 in File S1), with some sites having elevated %MeHg compared to that found in the literature (typically <1% MeHg). These sites with %MeHg >2 were mostly uncontaminated locations with varying %LOI. Similarly elevated %MeHg (>2%) has been measured in San Francisco Bay [27]. Sediment MeHg was also significantly correlated with sediment THg across sites (p = 0.002, n = 10). Sediment data collected across ten sites showed a significant positive linear relationship between %LOI and log-transformed THg (p = 0.039, n = 10; Figure 2), however, the relationship between %LOI and log MeHg (p = 0.17, n = 10; Figure 2) was not significant.

Water column THg concentrations ranged over an order of magnitude across sites for filtered concentrations (0.24–1.92 ng/L) and more than three orders of magnitude for particulate concentration (0.40–704.1 ng/g). Water column MeHg concentrations ranged over an order of magnitude for filtered concentrations (0.001–0.025 ng/L) and over two orders of magnitude for particulate concentrations (0.14–20.11 ng/g) (Table S1 in File S1). There was also a large variation in the %MeHg in the particulate fraction with three sites having low values comparable to the sediment (<2%) while most sites had much higher %MeHg (>10% MeHg). Across sites, there was a significant positive relationship between filtered MeHg and particulate MeHg (p = 0.0225, n = 9) but not for THg (p = 0.1683, n = 9).

The only statistically significant relationship between sediment and water compartments was the positive relationship between sediment total Hg and particulate total Hg concentrations (p = 0.025, n = 9). There were no significant relationships between MeHg in sediment and MeHg in filtered or particulate fractions. In the water column, there was no direct relationship between filtered THg or filtered or particulate MeHg concentrations and DOC, but there was a marginally significant relationship between particulate THg concentrations and DOC (p = 0.056, n = 8).

### Biota

**Figure 3:**
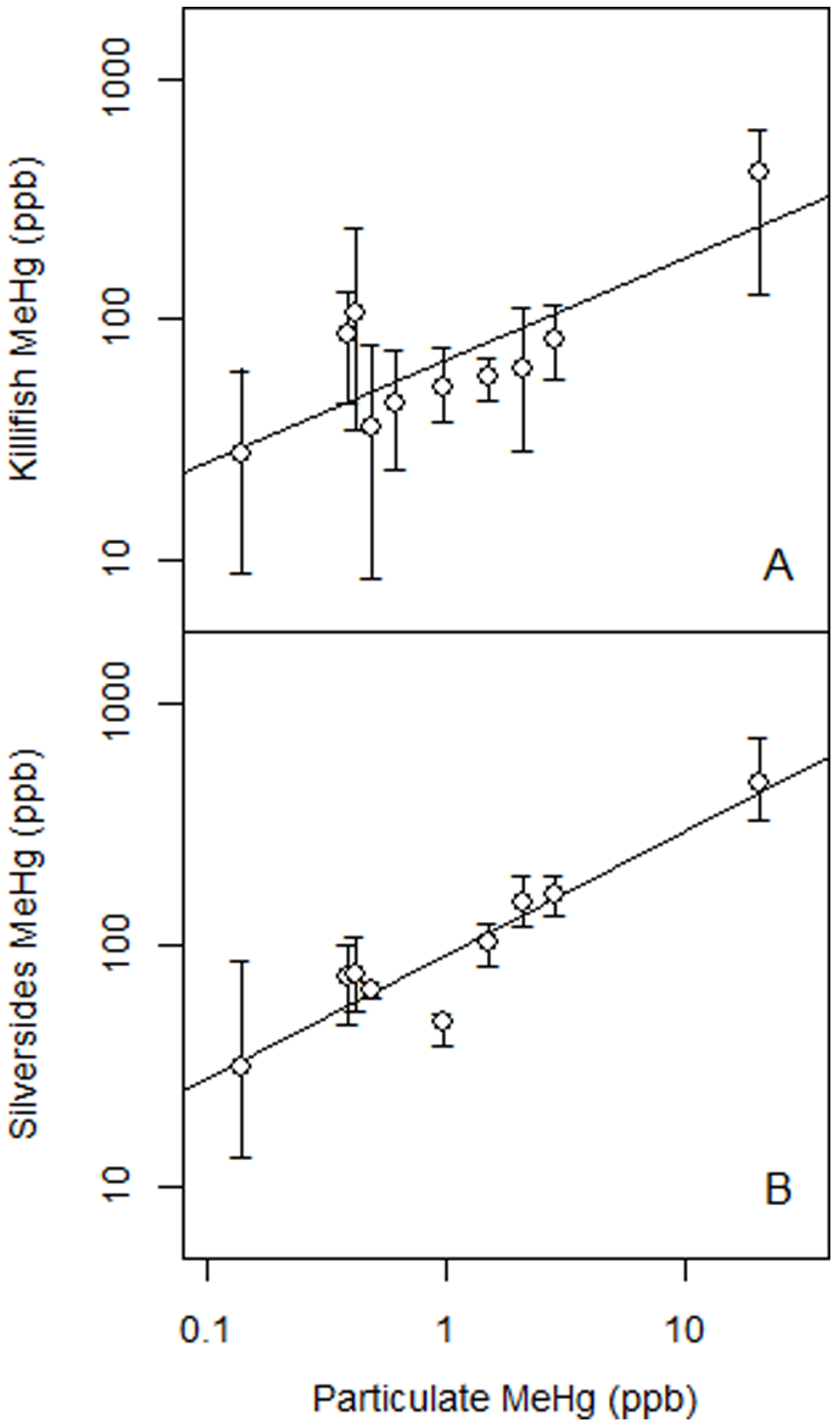
Relationship between MeHg tissue concentrations in fish and MeHg in water column particulate. Relationships between continuous variables assessed by linear regression after logarithmic transformations. (A) killifish MeHg vs. particulate; (B) Atlantic silverside MeHg vs. particulate. Points show site means and error bars extend to the minimum and maximum site concentrations.

**Figure 4:**
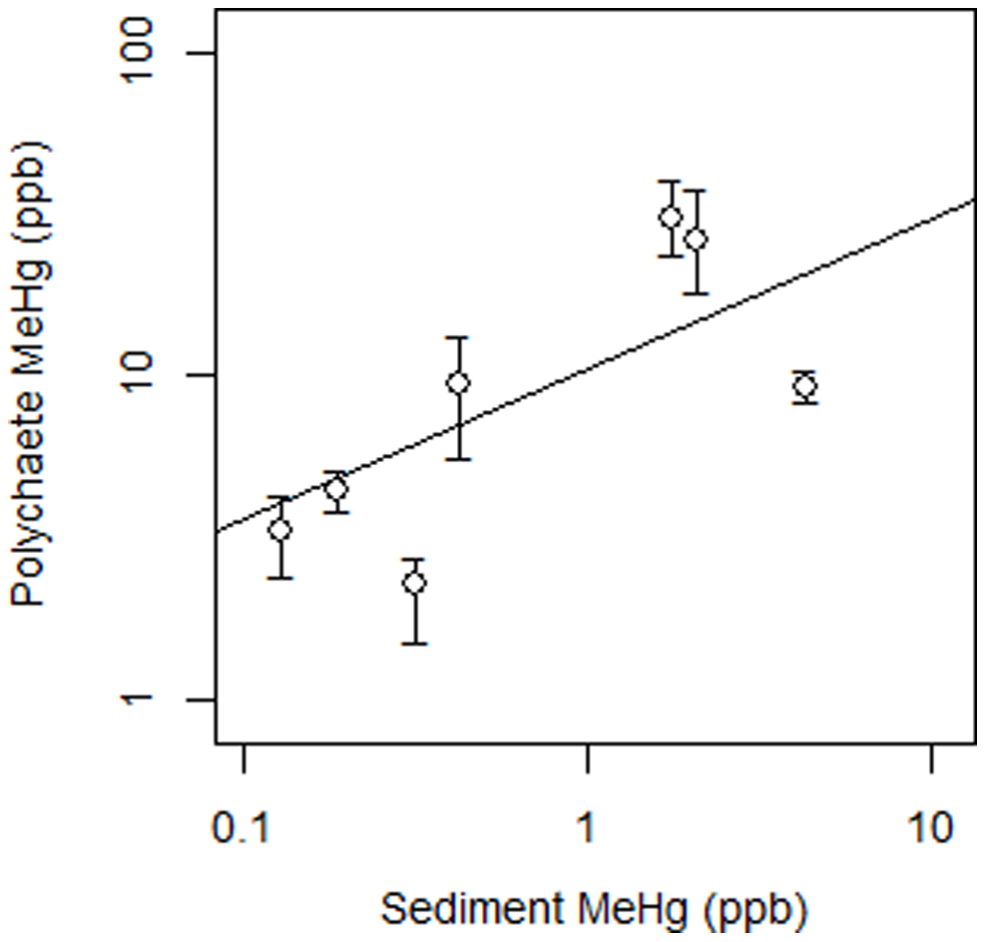
Relationship between MeHg in worm tissues vs. sediment MeHg. Relationships between continuous variables assessed by linear regression after logarithmic transformations. Points show site means and error bars extend to the minimum and maximum site concentrations.

Using multiple regression models to determine the variables best accounting for variation in MeHg concentrations in biota, we found that the concentrations in the different biota were associated with different sediment and water column compartments (Table 1; Table S2 in File S1). Specifically, MeHg concentrations in fish (killifish and silversides) showed significant positive associations with the corresponding water column particulate MeHg concentrations but not with filtered MeHg or sediment MeHg (Table 1; Figure 3). For the full model, sediment and water column variables accounted for 87% of the variation in killifish and, in the reduced model, particulate MeHg alone accounted for 62% of tissue concentration variation. Sediment and water column variables accounted for 92% of the variation in silverside MeHg concentrations, and particulate MeHg concentration alone accounted for 88% in the reduced model. MeHg concentrations of worms were related to sediment MeHg concentrations but not water column concentrations (Figure 4). In the full model, sediment and water column MeHg concentrations accounted for 69% of the tissue variation in worms and when only sediment MeHg was included in a reduced model, it accounted for 55% of the variation (Table 1). Sediment and water column MeHg concentrations were not significantly related to tissue concentrations in mussels or green crabs.

**Table 1:** Multiple regression results for environmental predictors (MeHg in sediments, water column dissolved aqueous, and particulates) of biotic MeHg tissue concentrations.

	Full Model			Reduced Model		
	Estimate	Std. Error	P-value	Estimate	Std. Error	P-value
	**A. Killifish**					
**Intercept**	2.169	0.435	0.004	1.837	0.067	<0.001
**log10(MeHg sediment)**	0.131	0.068	0.112	–	–	–
**log10(MeHg dissolved)**	0.150	0.186	0.457	–	–	–
**log10(MeHg particulate)**	0.27	0.135	0.060	0.424	0.117	0.007
**R^2^ (adj. R^2^)**	0.87 (0.79)			0.62 (0.57)		
**n**	9			10		
	**B. Silversides**					
**Intercept**	2.401	0.479	0.007	1.96	0.042	<0.001
**log10(MeHg sediment)**	0.011	0.070	0.878	–	–	–
**log10(MeHg dissolved)**	0.192	0.201	0.394	–	–	–
**log10(MeHg particulate)**	0.418	0.133	0.035	0.512	0.071	<0.001
**R2 (adj. R2)**	0.92 (0.86)			0.88 (0.87)		
**n**	8			9		
	**C. Worms**					
**Intercept**	0.604	0.921	0.559	1.02	0.119	<0.001
**log10(MeHg sediment)**	0.270	0.197	0.264	0.471	0.174	0.035
**log10(MeHg dissolved)**	−0.106	0.396	0.806	–	–	–
**log10(MeHg particulate)**	−0.312	0.337	0.423	–	–	–
**R2 (adj. R2)**	0.69 (0.37)			0.55 (0.47)		
**n**	7			8		

Full and reduced models for (A) killifish; (B) Atlantic silversides; and (C) worms.

### Food Web Attributes

**Figure 5:**
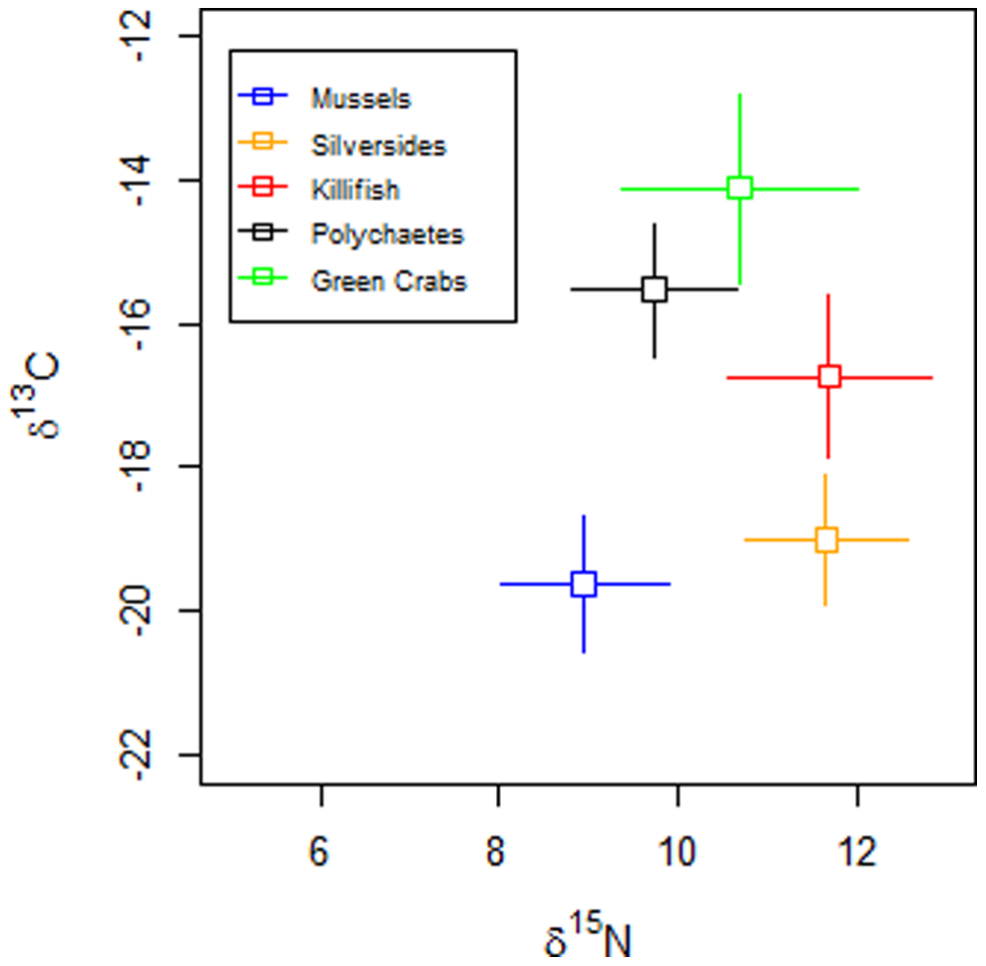
Stable isotope signatures of individual taxonomic groups measured as delta ^13^C and delta ^15^N. Delta ^15^N values were adjusted for site differences. Because of significantly different patterns, worms were excluded from analysis. Crosshairs show +/− two standard errors.

Measurements of stable isotopes as indicators of trophic level (δ^15^N) and carbon source of food (δ^13^C) were used to determine trophic and feeding relationships between fauna and their relationship to biotic MeHg concentrations. When adjusted for site, relative δ^15^N values indicate that the two fish species represented the highest trophic levels followed by green crabs, worms, and then mussels (Figure 5; Table S2 in File S1). Mussels and silversides were the most depleted in δ^13^C indicating their pelagic food sources whereas killifish consume food sources more intermediate between pelagic and benthic, and worms and green crabs had the least depleted δ^13^C indicating the most benthic food sources (Figure 5; Table S2 in File S1). Although two sites had blue mussels rather than ribbed mussels, the δ^13^C of the two species of mussels were not distinctly different and blue mussel values fell within the range of ribbed mussels.

**Figure 6:**
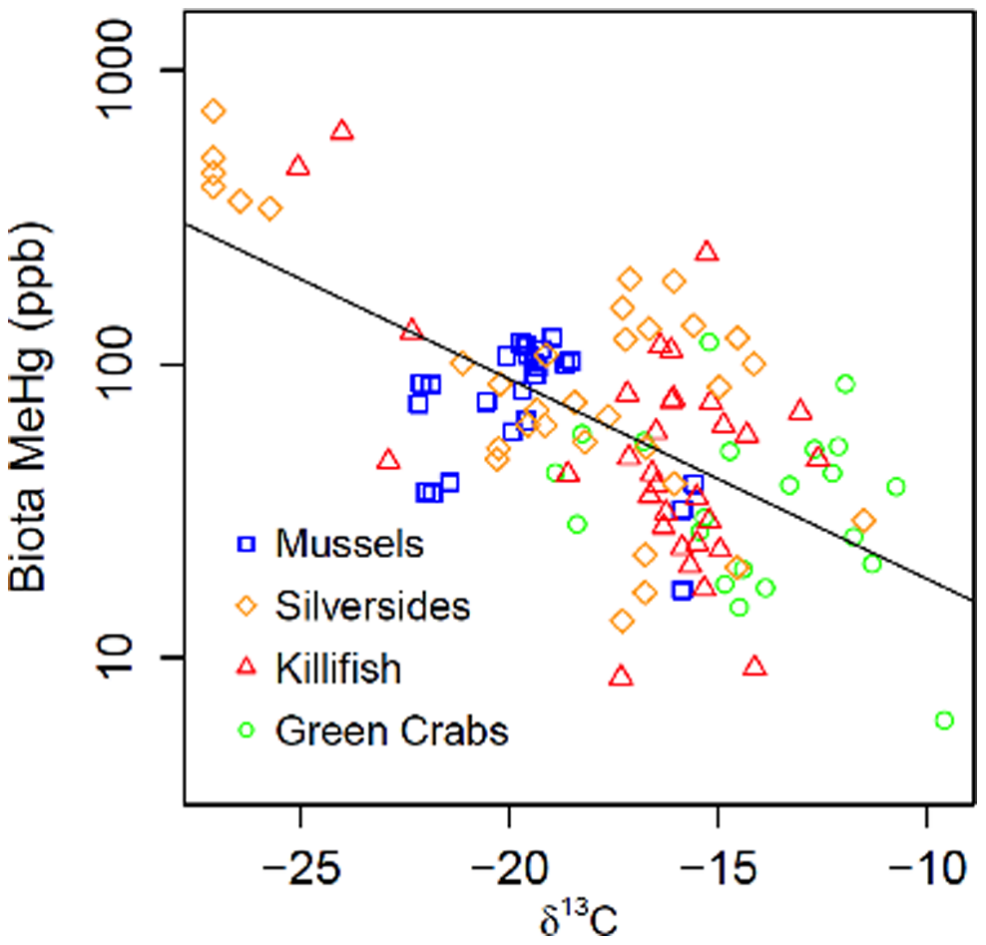
Relationship of MeHg tissue concentrations of all taxonomic groups across all sites vs. delta ^13^C. Relationships between continuous variables assessed by linear regression after logarithmic transformations.

Incorporating all biotic MeHg data to determine the relationship of bioaccumulation to food source, we found that across our range of estuarine sites, higher MeHg bioaccumulation was found in the more pelagic feeding (more δ^13^C depleted) organisms (Figure 6; Table S2 in File S1) (p = 0.00005, n = 32 species-location combinations). In fact, within killifish and Atlantic silversides, MeHg concentrations were higher at sites where individuals were more pelagic feeding (p = 0.001, n = 18 species-location combinations).

## Discussion

This study presents field data from a broad range of estuarine sites that differentiates the pathways of MeHg to bioaccumulation in a range of estuarine fauna. Our results show that MeHg in the water column but not in sediments is an important predictor for bioaccumulation in estuarine forage fish. MeHg in particulates is positively related to MeHg in transient (Atlantic silversides) and resident (killifish) fish. In contrast, sediment MeHg concentrations were only directly related to MeHg in deposit feeding infauna represented in this study by worms. This indicates that although sediments are the main repository for metal contaminants in estuaries, especially for Hg and MeHg, and a potential source of dissolved (and particulate) MeHg to overlying water, this benthic flux must not be directly related to sediment MeHg content, as has been shown by others, e.g. [43], [20]. Therefore, bulk sediments are not an accurate predictor of MeHg bioaccumulation in these forage fish. Alternatively, our sampling strategy may not have effectively captured the bioavailable sediment MeHg fraction although porewater concentrations at a subset of our sites are not related to water column concentrations (Balcom et al., unpublished data).

While the water column exposure to MeHg could still be the result of the complex coupling between water and sediment MeHg and *in situ* methylation in these shallow ecosystems, it could also potentially reflect differences across ecosystems in the influx of MeHg produced in the watershed or offshore which is subsequently taken up by the *in situ* estuarine phytoplankton. Sediment MeHg can be transferred to the water column through desorption from resuspended sediment or from the flux (diffusive and advective) of dissolved MeHg from sediments [62]. Although bulk total Hg and MeHg concentrations in sediments are much higher than in water, MeHg in sediments is not directly bioavailable to fish. Rather, the transport (flux) of MeHg from sediments into the water column is necessary for phytoplankton or particulate uptake and ingestion by invertebrates and fish. This may be a more important pathway than direct uptake from sediments for assimilation of MeHg produced in estuarine sediments into estuarine food webs [63]. Similarly, dissolved MeHg from upstream and offshore sources could be taken up into phytoplankton or benthic algae growing on the sediment surface. As discussed above, the relative importance of *in situ* production of MeHg versus external inputs varies across estuaries and coastal systems [11], [12], [19], [28].

Most sediment and water column total Hg and MeHg measurements for estuaries included in this study (Table S1 in File S1) are within the range of other east coast estuarine measurements [25], [76], [23], [40] (Balcom et al., unpublished data). Average sediment concentrations at Waquoit and Buzzards Bay (MA) were below the typical range for estuaries, but %MeHg (fraction of total Hg as MeHg) was within the range of values for the other sites in this study. Mill Creek on the Hackensack River was elevated in both average total Hg (2960 ng/g) and MeHg (34.8 ng/g), but total Hg was comparable to Hackensack River sites sampled in 2009 and earlier (Balcom et al., unpublished data; [19]). Surface water dissolved total Hg measurements (Table S1 in File S1) were within the range of values for NY/NJ Harbor [19] and Long Island Sound (LIS; [64], Balcom et al., unpublished data). While measured dissolved MeHg was low in the current study, and typically below levels measured previously in NY/NJ Harbor and LIS, the %MeHg was relatively uniform and within the range of other east coast estuarine measurements (Balcom et al., unpublished data, [29]). The %MeHg was comparable to other estuaries at about half the sites (Balcom et al., unpublished data), including the Hackensack River where particulate MeHg was high (20.1 ng/g). Water column THg was low compared to literature values at Waquoit [65], Barn Island [66], and Jamaica Bay [19] which resulted in elevated %MeHg at those sites. Nonetheless, the overall range of values in sediments and water column were representative of other systems in the region.

The importance of the water column as a source of MeHg to estuarine fauna has been examined in past studies, particularly for amphipods. Williams et al. [62] demonstrated that MeHg assimilation by amphipods via algal food was much greater than from dissolved aqueous exposure. Lawrence and Mason [37] performed mesocosm studies with amphipods to examine the relative importance of sediment versus water column particulate (algal additions) as a source of MeHg, From their experiments and modeled results they concluded that the sediment (particulate uptake and porewater exposure) was not an important source of MeHg except under conditions of highly contaminated sediments of low organic content, and low water column particulate MeHg. This scenario does not correspond to any of the ecosystems studied here, reinforcing the idea that direct uptake from the sediment is not likely the main source of the MeHg accumulating in the forage fish.

MeHg bioaccumulation is largely determined by movement and feeding mode of biota. Both killifish and mussels are biosentinel species of local Hg exposure given their small-scale patterns of movement in estuaries [59]. In contrast, Atlantic silversides are transient species and move in broader ranges throughout the estuary [59]. Mussels feed predominantly on suspended particles including phytoplankton while killifish derive much of their nutrition from the benthos and silversides are predominantly plankton consumers [13], [59]. Despite their different feeding strategies, MeHg bioaccumulation in both fish species is directly related to water column particulate MeHg. The percent of variation accounted for by MeHg in particulates is higher in silversides than killifish (88% vs. 62%) likely due to their more planktivorous feeding mode than for killifish which also feed on benthic food sources [59], [67]. Moreover, similar to our previous study [30] and other studies [68], [69], pelagic-feeding organisms (as determined by depletion in δ ^13^C) have higher concentrations of MeHg than benthic-feeding species, providing additional evidence that even in these shallow ecosystems, lower levels of the *pelagic* food web have an important role in transferring mercury to higher level marine organisms consumed by humans [30].

While the relationship of fish to water column MeHg particulate concentrations has not been previously documented in coastal marine systems, studies in both lakes and streams suggests that Hg and MeHg bioaccumulation is greatly influenced by the supply of Hg to the base of the pelagic food web [21], [22]. Chasar et al. [21] found that MeHg in fish and invertebrates in streams were strongly positively associated with filtered fractions of MeHg and DOC in surface water regardless of the trophic position of the organisms. In a study of lakes in the Great Lakes region, Rolfhus et al. [22] showed that bioaccumulation factors (BAF) and biomagnification factors (BMF) across 10 lakes were extremely similar, suggesting that the variation in fish concentrations was due to differences in aqueous supply of MeHg. Particulate MeHg concentrations were not considered or measured in either of these studies. Other studies in freshwater have also supported the conclusion that MeHg bioaccumulation in fish is driven more by MeHg uptake at the base of the food web than trophic factors such as trophic position of the fish or biomagnification rates [69], [70]. Fry and Chumchal’s [67] study of Hg in estuarine food webs found that stable isotope signatures for fish were similar to particulate organic matter suggesting a particulate source of food. In this study, we show directly the importance of MeHg in water column particulates to bioaccumulation and trophic transfer in fish.

In other estuarine field studies, links have been demonstrated between sediment and biotic Hg and MeHg concentrations. Gehrke et al. [31] concluded that Hg in forage fish (Missisippi silversides and topsmelt) in San Francisco Bay was derived from sediments based upon their related Hg stable isotope signatures. In Narragansett Bay RI, Taylor et al. [32] found a relationship between carbon-normalized THg in sediment and THg in zooplankton, invertebrates and finfish. In both of these studies, the relative importance of the aqueous pathway was not evaluated since water concentrations of Hg and MeHg were not measured. Indeed, in our previous work, sediment MeHg concentrations were shown to be related to biotic concentrations when all taxa are included in the analyses [30]. However, MeHg in aqueous fractions were also not measured in our earlier study.

Organic matter has an important role in controlling the biogeochemistry of MeHg in sediments and in the water column. In this study, as in others, there is a strong positive association between sediment THg and sediment organic matter (% LOI) [11], [23], [30], [38], [71]. This relationship is likely due to the binding of mercury to reduced sulfur groups [72]–[74] both within the water column (measured as DOC) and in the sediment [71] (measured as %LOI) and the positive relationship between organic matter and Hg inputs. In most other studies of individual systems, a linear relationship between THg and %LOI or sediment TOC, and between THg and MeHg, has been found [16], [25], [38], [75]–[78]. However, this is not always the case for MeHg and %LOI where the relationship was either weak or not significant, or varied seasonally [16], [40], [75], [76], [79], as found here.

The positive relationship between total Hg in sediments and water column particulates in this study suggests that resuspension of surface sediments may contribute a fraction of the particulate. In mesocosm resuspension experiments where TSS was up to 150 mg L^−1^, it was found that the particulate Hg in the water column was similar to that of the surface sediment, as expected [80], [81]. However, even with such high resuspension, the water column particulate MeHg was higher than that of the surface sediment, but had a low %MeHg (<1%). In the non-resuspended mesocosms, the particulate MeHg was three times higher, and the %MeHg was up to 6%, reflecting the fact the biota MeHg concentrations per gram are much higher than those of the surface sediment, and that MeHg is more bioaccumulative than inorganic Hg [80], [62]. Therefore, the lack of a relationship between MeHg in sediments and aqueous compartments suggests that MeHg in resuspended sediments is not the main contributor to MeHg in the particulate, and that bioaccumulation by particulates must be occurring. Although we have no data on particulate composition, the variable %MeHg in the particulate likely reflects the differences in sources, being lower in systems with relatively higher amounts of resuspension or abiotic particles. In addition, our data for pore water from a subset of these sites indicates that there is no direct relationship between water column concentrations and porewater concentrations suggesting that the two compartments are not tightly coupled, as has been found in other studies [39], [23] (Balcom unpublished data).

**Figure 7:**
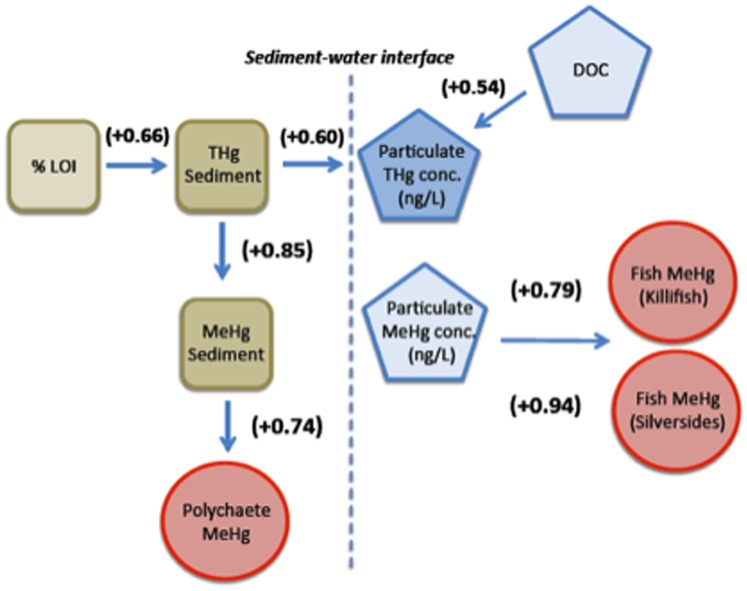
Relational diagram for total Hg and MeHg in sediment and water column and biotic compartments. The magnitude and sign of the coefficients represent the relative contribution of the independent variable in the prediction of the dependent variable located at the head of each arrow. These were calculated by multiplying the linear regression coefficients by the ratio of standard deviations of the independent and dependent variables, respectively.

Past studies on MeHg in estuarine sediments have examined the role of organic carbon quantity and quality in controlling MeHg production [23], [37]–[40], [76]. While these studies have suggested that sediment organic matter may limit Hg methylation in human impacted sites, a more recent study suggests that Hg methylation is greatest in sites with the highest Hg content (relative to carbon) and that organic matter is not the only factor influencing methylation. The level of sediment sulfur is also an important consideration. In the water column, studies on the relationship between DOC and Hg in freshwater also show a positive relationship indicative of the binding of Hg by DOC [82]–[85]. In our study, MeHg in sediment is not strongly related to TOC and DOC is only marginally related to MeHg in water column particulates, indicating that the relationships between carbon and MeHg in sediment and water column compartments are complex (Figure 7). Other factors, such as the rate of production of MeHg in sediments and the sediment binding capacity for MeHg influence the potential for sediments to be a source of MeHg to the overlying waters. As noted, DOC impacts the bioavailability of MeHg in the water column to plankton, and DOC could influence the rate of degradation of MeHg by photochemical processes within the water column. However, it is not just the amount but also the type and nature of the organic matter (e.g. fraction of reduced S present) that influences the fate, transport and bioaccumulation of MeHg in estuarine systems.

Therefore, the relationship between TOC or DOC and MeHg bioaccumulation in primary producers, primary consumers, and secondary consumers is not clear. In this study, there are no significant relationships between biotic concentrations and organic carbon (TOC or DOC). While some freshwater studies have shown a positive relationship between DOC and Hg concentrations in phytoplankton, zooplankton, invertebrates, and fish [82], [85], [86] others have found a negative relationship between DOC and Hg concentrations in phytoplankton and fish [87]. Negative relationships have also been found between DOC and Hg bioaccumulation factors (BAF) for freshwater algae, microseston, zooplankton and fish [35], [36], [82], [85]. In estuarine and marine systems, DOC has lower concentrations and lower molecular weights than in freshwater, and the DOC is derived more from *in situ* degradation and has less humic character, which may alter the biovailability of Hg and MeHg. Our past work and that of others showed negative relationships between TOC and Benthic-Sediment Concentration Factors for MeHg [30], [33], [37]. Others have found relationships in experimental marine studies where increased DOC has resulted in decreases in Hg uptake from water in marine amphipods, green mussels, and American oysters [37], [88]–[90]. However, most of these study results were based on aqueous exposures in the laboratory rather than bioaccumulation from ingestion in natural food webs.

Studies of coastal marsh food webs indicate that the primary consumers obtain nutrition from both benthic microalgae on the sediments and from phytoplankton and resuspended organic matter in the water column [13], [91], [92]. Higher trophic level organisms like fish and crabs accumulate most of their MeHg primarily through dietary sources [93], [94], whereas bivalves can take up mercury from water and sediment as well as dietary sources [89], [95]. The stable isotope signatures determined for different taxa in this study suggest that consumers are obtaining their MeHg from different pathways (Figure 7). While killifish are less pelagic feeding than Atlantic silversides, MeHg concentrations of both fish species are related to water column particulate MeHg concentrations. The killifish are likely consuming more from the benthic sources including resuspended organic matter (algae and detritus) and benthic crustaceans which have been shown to reflect water column concentrations [37], whereas the Atlantic silversides are feeding more on plankton [59,91 23,67]. Even though the crabs have higher δ^13^C signatures indicating a more benthic feeding strategy than worms, their tissue concentrations of MeHg are not related to sediment MeHg concentrations as are the worms. However, worms with their infaunal life histories and deposit feeding strategies are exposed directly to MeHg from ingestion of sediments and exposure to MeHg in pore water. Mussels are most depleted in δ^13^C indicating pelagic-feeding, but perhaps surprisingly there were no significant relationships between their tissue concentrations and sediment and water column MeHg concentrations.

Although sediments are the repositories of Hg in estuaries and sources of MeHg production, water concentrations of MeHg are the most direct predictor of MeHg in forage fish that are important prey for larger fish consumed by humans. MeHg in the water column may come from internal methylation in sediments or from external sources (watershed inputs or inputs from ocean currents) but the relative importance of these sources is currently poorly understood. For example, as discussed above, while sediments were found to be a minor source of MeHg to the water column of Passamaquoddy Bay, and the Gulf of Maine [11], [12], Mason et al. [11] concluded that about 60% of the MeHg accumulating in fish in the Chesapeake Bay was produced internally. Similarly, for the Hudson River estuary watershed, external inputs are the dominant source of MeHg, while *in situ* production is more important in Long Island Sound [20], [28]. Clearly, the overall characteristics of the estuary, such as depth, volume, tidal exchange and relative freshwater input, will have a large impact on the relative importance of sources of MeHg to the water column.

Identification of the predominant sources of MeHg to the water column pathway of MeHg exposure is complex and not clearly understood, but is important to both the management of estuarine sediments as well as to the development of models of MeHg fate in coastal food webs. Most contaminated sites are managed based upon sediment concentrations of Hg as opposed to water column concentrations. The results of this study suggest that water column exposure of estuarine fauna needs to be considered in assessments of contaminated sites. Models of estuarine and coastal food webs also require field-based understanding of MeHg pathways of trophic transfer. Thus, identifying the environmental sources of MeHg to estuarine food webs requires both an understanding of the biogeochemical factors influencing production and flux of MeHg from sediments into the water column and the influence of MeHg transport from external sources.

## Supporting Information

File S1 Supporting tables.
(DOCX)
