# Supplementary material for: Benthic and Pelagic Pathways of Methylmercury Bioaccumulation in Estuarine Food Webs of the Northeast United States
Source: PLoS One. 2014 Feb 18;9(2):e89305. doi: 10.1371/journal.pone.0089305 (PMC3928433; doi:10.1371/journal.pone.0089305)
Supplement: File S1 — Supporting tables. (DOCX) [file pone.0089305.s001.docx]

**SUPPORTING INFORMATION**

**Table S1.** Sediment and water (dissolved and particulate) mercury concentrations, sediment % LOI, water column DOC, and total suspended solids at each of ten sites on the US East Coast.

| **Site** | **sediment**  **Hg**  **(ng/g,dry)^a^** | **sediment MeHg**  **(ng/g,dry)** | | **sediment**  **%MeHg**  **(dry)** | **sediment %LOI** | **filtered**  **MeH g (ng/L)** | **particulate**  **MeHg (ng/g)** | **filtered THg (ng/L)** | **particulate THg (ng/g)** | **DOC (µg/g)** | **TSS (mg/L)** |
| --- | --- | --- | --- | --- | --- | --- | --- | --- | --- | --- | --- |
| Wells ME | 9.43 | 0.43 | 5.0 | | 2.4 | 0.001 | 0.14 | 0.24 | 16.65 | 22.58 | 35.3 |
| Portsmouth NH | 369.0 | 2.09 | 0.57 | | 5.0 | 0.005 | 0.39 | 0.71 | 10.03 |  | 31.2 |
| Waquoit MA | 3.20 | 0.06 | 2.3 | | 6.0 | 0.009 | 0.62 | 0.45 | 0.40 | 21.22 | 41.9 |
| Buzzards Bay MA | 5.70 | 0.13 | 2.4 | | 0.9 | 0.005 | 2.83 | 0.46 | 8.22 | 23.10 | 48.7 |
| Providence RI | 220.5 | 0.62 | 0.3 | | 5.4 | 0.002 | 0.97 | 0.43 | not analyzed | 19.60 | 55.6 |
| Barn Island CT | 73.8 | 0.19 | 0.3 | | 3.7 | 0.004 | 2.12 | 0.43 | 9.43 | 19.56 | 29.7 |
| Old Saybrook CT | 90.15 | 1.75 | 1.9 | | 5.2 |  | 0.42 | 0.38 | 25.07 | 22.45 | 30.8 |
| Milford CT | 244.5 | 4.33 | 1.8 | | 6.2 | 0.003 | 0.49 | 0.41 | 45.74 | 21.16 | 28.7 |
| Jamaica Bay NY | 201.5 | 0.32 | 0.2 | | 5.6 | 0.015 | 1.50 | 0.68 | 3.21* | 19.31 | 101.2 |
| Hackensack NJ | 2961.5 | 34.8 | 1.2 | | 16.2 | 0.025 | 20.11 | 1.92 | 704.07 | 27.84 | 27.7 |

**Table S2.** Mercury concentrations and stable isotope signatures in biota collected at each of ten sites on US East coast (n = 3).

| **Site** | **Species** | **MeHg ng/g (SD)** | **THg ng/g (SD)** | **% MeHg (SD)** | **d15N (SD)** | **d13C (SD)** |
| --- | --- | --- | --- | --- | --- | --- |
| Wells ME | Green crab | 25.98 (14.69) | 35.43 (16.89) | 71.47 (5.51) | 8.07 (1.02) | -14.87 (2.50 |
|  | Killifish | 27.71 (16.65) | 34.83 (16.07) | 77.76 (21.39) | 7.98 (1.87) | -15.39 (0.79) |
|  | Mussel | 73.37 (8.67) | 123.42 (14.35) | 59.44 (1.04) | 6.55 (0.37) | -19.95 (0.53) |
|  | Polychaete | 9.48 (3.31) | 56.15 (32.50) | 22.36 (12.86) | 8.73 (1.04) | -16.68 (1.13) |
|  | Silversides | 31.24 (27.10) | 42.95 (26.06) | 70.38 (21.54) | 8.03 (1.85) | -16.18 (2.93) |
| Portsmouth NH | Green crab | 76.58 (32.39) | 116.29 (32.39) | 62.91 (30.82) | 9.83 (0.77) | -12.97 (1.95) |
|  | Killifish | 86.90 (42.22) | 130.38 (29.82) | 67.53 (28.94) | 12.02 (0.79) | -18.68 (3.66) |
|  | Mussel | 105.44 (11.04) | 257.35 (6.89) | 41.07 (5.46) | 7.23 (0.25) | -19.49 (0.22) |
|  | Polychaete | 26.04 (9.91) | 161.33 (30.39) | 16.46 (6.08 | 10.43 (0.32) | -13.70 (0.47) |
|  | Silversides | 74.00 (26.57) | 84.37 (28.26) | 87.63 (6.59) | 11.92 (0.83) | -19.95 (1.38) |
| Waquoit MA | Green crab | 41.21 (11.24) | 49.40 (11.24) | 82.96 (4.35) | 7.66 (0.98) | -13.43 (1.67) |
|  | Killifish | 44.54 (26.05) | 46.63 (27.00) | 95.27 (0.63) | 8.26 (2.07) | -15.40 (0.20) |
|  | Mussel | 37.54 (1.69) | 49.39 (8.70) | 77.57 (13.75) | 10.29 (0.23) | -21.76 (0.30) |
|  | Polychaete | 4.02 (1.14) | 30.05 (10.25) | 15.054 (7.54) | 7.22 (0.11) | -13.51 (1.19) |
| Buzzards Bay MA | Green crab | 38.66 (12.22) | 45.92 (12.22) | 83.54 (3.86) | 7.31 (0.72) | -14.48 (1.09) |
|  | Killifish | 82.79 (29.15) | 94.94 (28.25) | 86.76 (8.61) | 10.23 (1.30) | -16.33 (0.19) |
|  | Mussel | 101.16 (10.23) | 163.03 (21.84) | 63.00 (12.64) | 8.45 (0.08) | -19.10 (0.38) |
|  | Polychaete | 3.34 (1.25) | 45.87 (4.43) | 7.44 (3.44) | 8.51 (3.42) | -14.93 (2.04) |
|  | Silversides | 160.58 (28.54) | 169.96 (25.81) | 94.30 (3.95) | 10.61 (0.55) | -16.32 (0.88) |
| Providence RI | Green crab | 39.43 (21.92) | 48.18 (21.92) | 79.76 (7.14) | 13.25 (0.30) | -17.33 (2.18) |
|  | Killifish | 52.33 (20.63) | 54.99 (21.23) | 95.00 (1.08) | 15.70 (0.09) | -16.38 (0.27) |
|  | Mussel | 94.78 (32.67) | 151.49 (35.34 | 62.17 (15.61) | 7.26 (0.52) | -19.15 (0.72) |
|  | Silversides | 47.56 (7.33) | 53.59 (8.95) | 88.98 (6.40) | 13.96 (0.76) | -17.68 (2.26) |
| Barn Island CT | Killifish | 62.80 (42.97) | 69.02 (45.00) | 89.80 (2.83) | 8.87 (1.11) | -16.16 (0.96) |
|  | Mussel | 109.74 (4.83) | 207.59 (15.46) | 53.03 (3.80) | 7.37 (0.27) | -19.75 (0.28) |
|  | Polychaete | 4.41 (0.59) | 12.84 (8.57) | 52.55 (42.44) | 7.21 (0.28) | -15.89 (0.50) |
|  | Silversides | 149.30 (39.78) | 156.03 (38.96) | 95.45 (1.47) | 10.05 (0.81) | -17.00 (0.30) |
| Old Saybrook CT | Killifish | 105.05 (114.76) | 120.68 (117.91) | 80.00 (10.84) | 11.27 (2.14) | -16.84 (1.67) |
|  | Polychaete | 30.33 (8.61) | 104.85 (71.17) | 33.79 (11.12) | 9.32 (0.31) | -19.74 (0.36) |
|  | Silversides | 75.90 (27.78) | 93.50 (25.03) | 79.94 (9.31) | 11.54 (0.57) | -18.32 (0.75) |
| Milford CT | Killifish | 36.00 (37.63) | 40.66 (39.13) | 81.81 (14.21) | 13.52 (1.23) | -16.73 (0.92) |
|  | Mussel | 81.27 (7.00) | 118.89 (14.75) | 69.45 (13.62) | 10.73 (0.33) | -22.06 (0.15) |
|  | Polychaete | 9.27 (0.98) | 71.63 (11.59) | 13.11 (1.95) | 11.88 (0.34) | -15.27 (0.25) |
|  | Silversides | 64.44 (4.53) | 81.95 (8.49) | 79.26 (10.51) | 14.21 (0.67) | -19.35 (0.20) |
| Jamaica Bay NY | Green crab | 32.23 (30.39) | 47.92 (30.39) | 60.50 (23.15) | 11.24 (1.12) | -10.81 (1.27) |
|  | Killifish | 57.71 (10.54) | 66.48 (5.31) | 86.74 (13.12) | 13.82 (1.24) | -13.32 (0.89) |
|  | Mussel | 29.29 (11.29) | 68.13 (10.98) | 44.99 (20.80) | 10.44 (0.01) | -15.77 (0.17) |
|  | Polychaete | 2.27 (0.65) | 37.165 (9.16) | 6.34 (2.47) | 10.53 (0.39) | -13.17 (0.67) |
|  | Silversides | 102.01 (19.70) | 111.50 (19.04) | 91.26 (2.02) | 13.97 (0.81) | -14.55 (0.41) |
| Hackensack NY | Killifish | 403.11 (248.83) | 421.98 (256.81) | 95.28 (1.99) | 14.17 (0.50) | -23.81 (1.38) |
|  | Silversides | 432.19 (69.29) | 501.08 (52.59) | 85.99 (7.18) | 11.44 (0.20) | -26.43 (0.68) |
